# Changes of Atherosclerotic Plaque in Cerebral Artery Stenosis According to High-Resolution MR Imaging

**DOI:** 10.3390/tomography8040141

**Published:** 2022-06-27

**Authors:** Min Soo Baek, Kang Hoon Lee, Seong Yoon Cho, Yong-Jin Im, Byoung-Soo Shin, Hyun Goo Kang

**Affiliations:** 1 Medical School, Jeonbuk National University, Jeonju 54907, Korea; mins527@naver.com (M.S.B.); hoon9893@daum.net (K.H.L.); apollon729@naver.com (S.Y.C.); 2 Center for Clinical Pharmacology, Biomedical Research Institute, Jeonbuk National University Hospital, Jeonju 54907, Korea; yjim@jbcp.kr; 3 Department of Neurology, Medical School, Jeonbuk National University Hospital, Jeonju 54907, Korea; sbsoo@jbnu.ac.kr; 4 Research Institute of Clinical Medicine of Jeonbuk National University—Biomedical Research Institute of Jeonbuk National University Hospital, Jeonju 54907, Korea

**Keywords:** atherosclerosis, magnetic resonance imaging, plaque, stroke

## Abstract

Atherosclerosis can affect multiple arteries, and result in stroke and heart disease. Clinical and conventional imaging is insufficient to predict the progression of atherosclerosis. This study investigates risk factors that rely on high-resolution magnetic resonance imaging (HR-MRI). Patients with cerebral artery stenosis who had undergone HR-MRI at least twice were included. The demographics, risk factors, and proportion of patients with cerebral artery stenosis were investigated. The association between atherosclerotic plaque characteristics and the progression or regression of artery stenosis was also analyzed. A total of 42 patients were analyzed, with a median follow-up of 16.88 ± 12.53 months. The mean age of all subjects was 63.1 ± 9.15 years, and 83.3% of them were male. The incidences of stenosis of the basilar, proximal internal carotid, and middle cerebral arteries were 21.4%, 61.9%, and 16.7%, respectively. Intraplaque hemorrhage (IPH) was detected in 20 (47.6%) patients. Multivariate analysis showed that age (odds ratio (OR), 0.87; *p* = 0.014), smoking (OR, 0.11; *p* = 0.033), and IPH regression (OR, 10.13; *p* = 0.027) were associated with stenosis regression. The progression of IPH (OR, 115.80; *p* = 0.007) was associated with stenosis progression. Results suggest that IPH on HR-MRI is associated with changes in cerebral atherosclerotic stenosis.

## 1. Introduction

Arteriosclerosis is one of the main causes of cerebral infarctions. It results in plaque formation under the intima wall of the artery, which gradually narrows the inner diameter of the blood vessels. Plaque stability and burden are important factors that can induce cerebral infarction. Cai et al. demonstrated that high-resolution magnetic resonance imaging (HR-MRI) could accurately classify human carotid atherosclerotic plaques according to the American Heart Association [1].

Cerebral embolism caused by atherosclerotic plaques or thrombosis occurring at the site of plaque rupture can lead to ischemic stroke and transient ischemic attack (TIA). Consequently, the accurate identification of the components and status of these atherosclerotic plaques through imaging is critical for predicting the risk of ischemic stroke. Many recent studies have reported that plaque characteristics can be used as major factors in predicting the development of ischemic stroke [2].

Apart from helping medical personnel in identifying the cause of cerebrovascular stenosis in the patient, HR-MRI also provides pathoecological information on the components of the atherosclerotic plaque. Most previous studies on HR-MRI have compared luminal stenosis and plaque burden in symptomatic and asymptomatic patients, and only a few studies have examined the changes in plaque components with time and the factors affecting them.

This study analyzes the components of atherosclerotic plaques by using HR-MRI in patients with cerebral arterial stenosis. Further, we aimed to understand the changes in the components of the plaque over time and their association with risk factors for atherosclerosis.

## 2. Materials and Methods

### 2.1. Study Population

This study selected 800 patients who had been admitted to the Department of Neurology of regional university hospitals between May 2012 and August 2021 for various symptoms (e.g., headache, stroke, and dizziness), diagnosed with intra- or extracranial artery stenosis, and then underwent HR-MRI. All patients underwent standard brain MRI and magnetic resonance angiography (MRA) to detect brain parenchymal lesions and to differentiate their causes. Patients who met one of the following conditions were excluded: (1) HR-MRI performed once, (2) arterial dissection, and (3) insufficient data. Dissection was defined as wall thickening with low signal intensity due to an intimal flap on black-blood (BB) T2-weighted imaging and high signal intensity due to an intimal flap on time-of-flight MR angiography (TOF-MRA).

### 2.2. Clinical Characteristics

The basic personal information of the patients was collected, such as age, sex, and the interval between HR-MRI measurements. Stroke history was defined as previous stroke onset before hospitalization, including ischemic and hemorrhagic strokes. This study investigates the administration and extent of antiplatelet agents and statins. Antiplatelet treatment was classified into mono- and dual antiplatelet therapy. Statin intensity was classified into three groups according to the 2019 ACC/AHA guidelines for the primary prevention of cardiovascular disease [3]. Further, the risk factors for atherosclerosis, including hypertension, diabetes mellitus, and dyslipidemia, were evaluated. Hypertension was defined as when hypertension was already diagnosed, when patients were taking antihypertensive drugs, or when patients showed persistent systolic blood pressure of 140 mmHg or more, or diastolic blood pressure of 90 mmHg or more when measuring blood pressure at rest. Diabetes mellitus was defined as when a patient had been previously diagnosed with diabetes mellitus and was taking a hypoglycemic agent or insulin, when a patient had a fasting blood glucose level of 126 mg/dL or higher, or a blood glucose level of 200 mg/dL or higher two hours after a meal at the time of admission. Uncontrolled diabetes was determined on the basis of the 2021 American Diabetes Association (ADA) criteria. It was defined as fasting plasma glucose (FPG) ≥ 126 mg/dL, 2 h plasma glucose ≥ 200 mg/dL, HbA1c ≥ 6.5% even under appropriate treatment, or in a patient with classic symptoms of hyperglycemia or hyperglycemic crisis who had a random plasma glucose level ≥ 200 mg/dL [4]. Dyslipidemia was defined as those patients who had been diagnosed with dyslipidemia in the past and were taking statins or those who satisfied the following criteria according to the 2019 ACC/AHA guidelines on the primary prevention of cardiovascular disease at the time of admission: (1) low-density lipoprotein cholesterol (LDL-C) ≥ 160 mg/dL; (2) high-density lipoprotein cholesterol (HDL-C) <40 mg/dL in men; <50 mg/dL in women; or (3) triglycerides ≥ 175 mg/dL [3]. Coronary artery disease was confirmed through electrocardiogram, exercise stress test, stress nuclear medicine myocardial perfusion imaging, and echocardiography to examine the previous diagnosis of angina, myocardial infarction, and suspected symptoms. Atrial fibrillation was defined as a history of atrial fibrillation or AF confirmed by an electrocardiogram. Additionally, smoking status and alcohol consumption at the time of admission were investigated. This study measured hemoglobin (Hb), white blood cell (WBC), platelet (PLT), hemoglobin A1c (HbA1c), fibrinogen degradation products (FDP), D-dimer, blood urea nitrogen (BUN), creatinine, glomerular filtration rate (GFR), uric acid, total cholesterol, triglycerides, high-density lipoprotein (HDL), and low-density lipoprotein (LDL) levels using laboratory tests.

### 2.3. Magnetic Resonance Imaging (MRI)

MRI was performed using a 3.0 Tesla MRI scanner (Achieva; Philips Medical Systems, Amsterdam, Netherlands) with a 16-channel head coil. TOF-MRA for delineating the lumen shape and plaque morphology was obtained in the axial plane, and the data were reconstructed using a dedicated online postprocessing tool to determine blood vessel architecture. Multimodal sequences of HR-MRI, including BB T1-weighted, BB T2-weighted, TOF axial, magnetization-prepared rapid acquisition with gradient echo (MPRAGE), and contrast-enhanced BB T1-weighted imaging, were obtained. BB T1-weighted and T2-weighted imaging was performed to determine the site of stenosis, which was confirmed using three-dimensional (3D) TOF-MRA. T1-weighted imaging was performed using a two-dimensional turbo spin-echo sequence under the following conditions: repetition time/echo time, 800/10 ms; field of view, 140 × 140 mm; matrix size, 140 × 150; slice thickness, 2.0 mm; echo train length, 10; number of excitations, 2. T2-weighted HR-MRI scans also used a turbo spin-echo sequence with the following conditions: repetition time/echo time, 3100/80 ms; field of view, 140 × 140 mm; matrix size, 140 × 140; slice thickness, 2.0 mm; echo train length, 20; number of excitations, 2. The imaging parameters for the TOF- MRA scan were as follows: repetition time/echo time, 18/3.8 ms; flip angle, 16°; field of view, 140 × 140 mm; matrix size, 312 × 165; slice thickness, 1.0 mm; echo train length, 1; number of excitations, 3. For 3D MPRAGE sequencing, the segment was acquired using sequence repetition time, inversion preparation time, and phase encoding order obtained from the MPRAGE sequences. The image parameter settings were as follows: repetition time, echo time, and inversion time, 8.8, 5.3, and 304 ms; flip angle, 15°; echo train length, 32; field of view, 140 × 140 mm; matrix, 216 × 198. The BB technique based on preregional 80 mm thick saturation pulses that saturates incoming arterial flow was used for all scans. Gadodiamide (0.1 mmol/kg body weight; Dotarem; Guerbet, Aulnay-sous-Bois, France) was administered intravenously to all patients before contrast-enhanced BB T1-weighted imaging. Contrast-enhanced BB T1-weighted imaging was performed approximately 5 min after contrast injection. The longitudinal coverage of each artery was 22–24 mm. Each scan lasted 3–4 min. The total scan time was between 25 and 30 min, and the patients remained in the MR machine for 35–45 min. Clinical diffusion-weighted imaging (DWI) and fluid-attenuated inversion recovery (FLAIR) images were used to confirm the infarct.

### 2.4. Image Analysis

Cerebral artery stenosis was classified as intracranial or extracranial artery stenosis according to location, and it was categorized as symptomatic or asymptomatic artery stenosis according to concomitant symptoms. Symptomatic stenosis was defined as a case in which stenosis was confirmed in a blood vessel concomitant with neurological symptoms.

**Figure 1:**
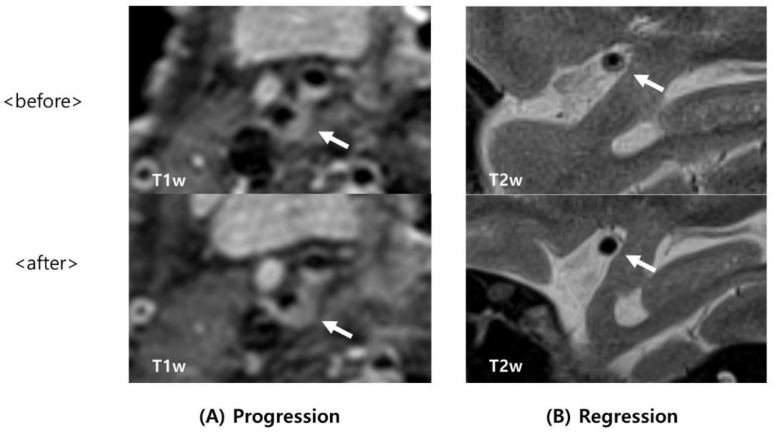
Examples of progression and regression of cerebral artery stenosis on HR-MRI. (**A**,**B**): Atherosclerotic plaque (arrow). (**A**) Upper panel: initial T1-weighted (T1w) HR-MR imaging reveals moderate stenosis in the right ICA; lower panel: follow-up T1w HR-MR imaging indicates severe stenosis in the right ICA. (**B**): Upper panel: initial T2-weighted (T2w) HR-MR imaging of the MCA with stenosis. Lower panel: follow-up T2w HR-MR imaging shows the MCA without stenosis. HR-MRI = high-resolution magnetic resonance imaging; ICA = internal carotid artery; MCA = middle cerebral artery.

**Figure 2:**
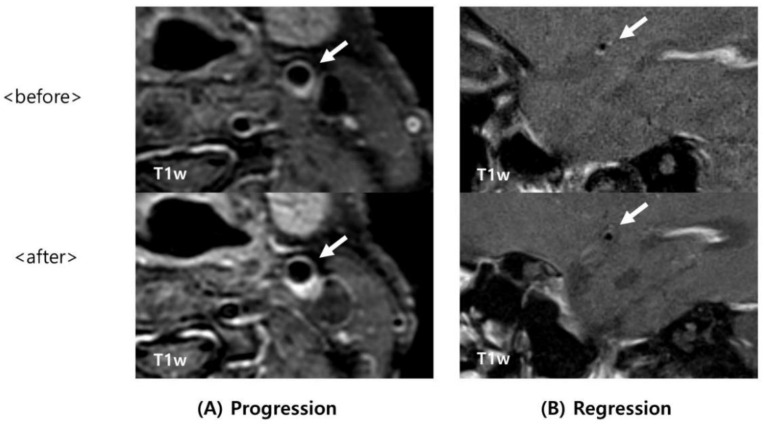
Examples of progression and regression of intraplaque hemorrhage on HR-MRI. (**A**,**B**) Atherosclerotic plaque with intraplaque hemorrhage (arrow). (**A**) Upper panel: initial T1-weighted (T1w) HR-MR imaging in the left CCA shows an eccentric plaque with intraplaque hemorrhage (IPH); lower panel: follow-up T1w HR-MR imaging shows significant atherosclerotic lesion with high signal intensity in the same area that indicates progression of IPH more than the initial imaging does. (**B**): Upper panel: initial T1w HR-MR imaging shows an eccentric lesion with IPH in the MCA. Lower panel: follow-up T1w HR-MR imaging reveals the MCA without IPH. HR-MRI = high-resolution magnetic resonance imaging; CCA = common carotid artery; MCA = middle cerebral artery.

Plaque was defined as a thickened focal wall rather than image slices obtained from below or above the focal wall on T1- and T2-weighted images. A region showing high signal intensity in atherosclerotic plaques was defined as an intraplaque hemorrhage (IPH). IPH was defined as high signal intensity on MPRAGE when it showed 200% more signal intensity than that of the surrounding tissue. Arterial dissection was carefully excluded on the basis of imaging features because a hyperintense signal could be observed in the intraluminal thrombus or hematoma in dissection or on precontrast T1-weighted images. Two researchers (Kang Hoon Lee and Seong Yoon Cho) who had not been informed about the clinical details performed analyses of HR-MRI, and classified patients into regression and progression groups (Figure 1 and Figure 2). As their interpretations of the three cases differed, a researcher (Byoung-Soo Shin) who is an experienced HR-MRI reader performed the final analysis.

### 2.5. Statistical Analyses

The demographics, risk factors, and proportion of patients with cerebral artery stenosis were assessed. The Pearson’s chi-squared, Fisher’s exact, Student’s *t*-, and Wilcoxon rank-sum tests were used for categorical or continuous variables as appropriate. Univariate and multivariate analysis models were used to investigate the factors associated with the regression and progression of cerebral artery stenosis. To avoid variable selection due to spurious correlations, only variables showing a potential association (*p* < 0.1) in the univariate analysis were included in the multivariate logistic regression model. A two-sided *p*-value < 0.05 was considered to be statistically significant. All statistical analyses were performed using Statistical Package for the Social Sciences version 21 (International Business Machines Corp., Armonk, NY, USA).

## 3. Results

**Figure 3:**
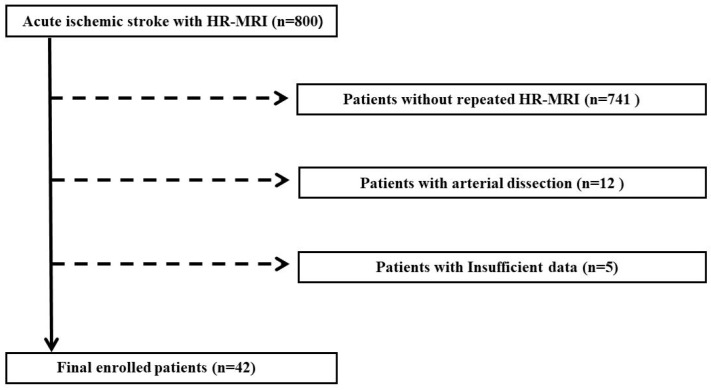
Flowchart of the study population. In total, 800 patients with acute ischemic stroke who had undergone HR-MRI were selected in this study. Analysis of HR-MRI was performed for the cerebral arteries of 59 patients who had undergone repeated HR-MRI. Twelve patients with arterial dissection and five patients with insufficient data were excluded from this study. This study lastly comprised 42 patients with atherosclerosis. HR-MRI, high-resolution magnetic resonance imaging.

During the study period, 800 patients with acute ischemic stroke underwent HR MRI at least once. Among them, 741 patients did not undergo HR-MRI twice or more, 12 patients had arterial dissection, and 5 patients had insufficient data. Consequently, 758 PATIENTS were excluded, and 42 patients were analyzed (Figure 3). The mean age of all 42 subjects was 63.1 ± 9.15 years, and 83.3% were male (Table 1). The interval of plaque MRI was 16.88 ± 12.53 months. Of the patients, 9 (21.4%) had basilar artery stenosis, 26 (61.9%) had proximal internal carotid artery (ICA) stenosis, and 7 (16.7%) had middle cerebral artery (MCA) stenosis. Of the patients, 16 (38.1%) had symptomatic stenosis, and IPH was observed in 20 (47.6%) patients. Four patients (9.5%) did not take antiplatelet drugs, and two patients (4.8%) did not take statins because the patients did not follow up in the middle, had a bleeding problem, or it was not applicable to the symptom. Table 1 summarizes the details of the baseline characteristics of the patients, including vascular risk factors and laboratory findings.

This study compared the characteristics of cerebral artery stenosis with and without the regression (Table 2). The mean age of stenosis with regression was significantly less than that of stenosis without regression (58.40 ± 7.43 vs. 65.70 ± 9.08, *p* = 0.011). Stenosis without regression cases had a very low ratio of MCA, whereas stenosis with regression cases had a very high ratio of MCA (*p* = 0.009). For this reason, stenosis cases with regression had a higher proportion of intracranial artery stenosis. The ratio of IPH inside the plaque, confirmed by HR-MR, was not significantly different between the two groups; however, the regression of IPH was observed significantly more in patients with stenosis with regression (40.0% vs. 11.1%, *p* = 0.029). When the risk factors for atherosclerosis were compared, the smoking percentage of patients with stenosis without regression was significantly higher than that of patients with stenosis with regression (44.4% vs. 13.3%, *p* = 0.04). Initial HbA1c and follow-up HbA1c were not significantly different in patients with stenosis without regression, but follow-up HbA1c was significantly higher than the initial HbA1c in patients with stenosis with regression (−1.60 ± 1.05 vs. 0.4 ± 1.34, *p* = 0.004).

Additionally, this study compared the characteristics of cerebral artery stenosis with and without disease progression (Table 3). The mean age of stenosis with progression was significantly greater than that of stenosis without progression (69.29 ± 6.65 vs. 61.86 ± 9.14, *p* = 0.048). HR-MRI revealed that patients with stenosis with progression had significantly more IPH than those with stenosis without progression (100% vs. 37.1%, *p* = 0.003). Further, progression of IPH was significantly higher in patients with stenosis with progression than in those without progression (85.7% vs. 2.9%, *p* < 0.001). The risk factors for atherosclerosis were not significantly different between the two groups. It was observed that the HbA1c of patients with stenosis without progression significantly decreased from initial to follow-up, while HbA1c of patients with stenosis with progression significantly increased from initial to follow-up (1.32 ± 1.49 vs. −0.93 ± 1.13, *p* = 0.003). 

On the basis of these results, multivariate analysis was conducted on variables related to the regression and progression of stenosis. Among the clinical and laboratory variables, age (odds ratio (OR), 0.87 [0.79–0.97]; *p* = 0.014), regression of IPH (OR, 10.13 [1.31–78.57]; *p* = 0.027), and smoking (OR, 0.11 [0.01–0.83]; *p* = 0.033) were significantly different between stenosis with and without regression. The results of the multivariate analysis show that the progression of IPH (OR, 115.80 [3.77–3554.18], *p* = 0.007) was significantly associated with the progression of cerebral artery stenosis (Table 4).

## 4. Discussion

Recent studies have reported that vulnerable plaques are an important predictor of atherothrombotic stroke [2]. Most previous studies on HR-MRI compared plaque features between symptomatic and asymptomatic patients [5,6,7,8]. Although Shi et al. [9] recently measured the progression of plaque burden using HR-MRI, they mainly focused on plaque burden and volume rather than plaque features, and only evaluated patients with intracranial atherosclerosis. The present study analyzed images of patients with cerebral artery stenosis who were followed up using HR-MRI at appropriate time intervals. We divided the subjects into an extracranial artery group and an intracranial artery group, and retrospectively analyzed the differences among patients with no change in cerebral artery stenosis, patients with progression, and patients with regression and factors affecting progression and regression.

Results show that the ratio of proximal ICA stenosis (extracranial artery stenosis, ECAS) was high, yet a considerable number of intracranial artery stenoses (ICAS) were also observed (61.9 vs. 38.1%, Table 1). In autopsy studies, IPH was found much less frequently in intracranial atherosclerotic plaques than in extracranial atherosclerotic plaques [10]. Compared with the extracranial artery, the intracranial artery has thinner media and adventitia, and a less-developed vasa vasorum. Therefore, intracranial plaques are less capable of undergoing neoangiogenesis during plaque progression [11]. Consequently, IPH may be less likely to occur in intracranial arteries than in extracranial arteries [11]. In this study, the proportion of patients with IPH was 47.6%, the frequency of IPH in patients with ECAS was 46.2%, and the frequency of IPH in patients with ICAS was 50%, which disagreed with the results of previous studies revealing that the frequency of IPH in patients with ICAS was lower. This difference could have occurred because of a smaller sample in the present study.

In the imaging features of atherosclerotic plaques, elements such as a fibrous cap, calcification, lipid or necrotic core, hemorrhage, and fibrosis could be found. Components influencing plaque vulnerability include the lipid core and IPH. As the plaque progresses, the volume of the lipid core increases, and the fibrous cap becomes thinner. When the volume increases further, neovascularization occurs inside the plaque to induce IPH [12]. When atherosclerotic plaques have surface defects, bleeding, or thrombi, they are considered to be vulnerable plaques. Cai et al. classified these plaques as Type VI in their study using HR-MRI [1]. Kolodgie et al. conducted a histopathological study using coronary artery specimens and argued that IPH acted as a potent atherogenic stimulus because it contributed to the accumulation of free cholesterol, macrophage infiltration, and the enlargement of a necrotic core [13]. Furthermore, Takaya et al. showed that IPH accelerates plaque progression in the carotid artery [14]. The results of the present study also confirm that IPH might be related to plaque progression, which is concordant with the results of previous studies on BA stenosis [15]. In addition, in our study, we could consider that IPH affects plaque regression.

Cigarette smoking is a risk factor for atherosclerotic disease, and is associated with carotid artery wall thickening and stenosis [16]. The results of our study confirmed that smoking had an adverse effect on cerebral artery stenosis. Smoking causes platelet activation and thrombogenesis in atherosclerotic plaques [17]. Our data further support the role of current smoking in atherogenic processes. Fasting glucose and HbA1c are independent risk factors for ICAS [18]. Moreover, Sun et al. [19] recently reported that an elevated HbA1c could be a risk factor of poor vulnerability of carotid plaque. Similarly, the results of our study show that both plaque progression and regression are associated with changes in HbA1c levels. However, we were unable to conduct multivariate analysis because the number of patients for whom HbA1c was measured was too small. Future studies examining a larger number of patients on the basis of the results of this study are required to further clarify whether the increase or decrease in HbA1c affects the progression and regression of plaques. 

The present study had some limitations. First, this study did not perform quantitative analyses of plaque features, such as plaque burden or volume, and the presented plaque characteristics were the result of qualitative analyses. This was because our study evaluated the factors influencing progression and regression rather than focusing on plaque features. To clarify the qualitative analyses, two researchers and an experienced HR-MRI reader performed the analyses of HR-MRI. Additional studies may address the quantitative changes in plaque features in cerebral artery stenosis. Second, although this study attempted to analyze plaque characteristics, such as the necrotic core and thin fibrous cap, suggesting unstable plaque, only IPH was observed because the diameters of the BA or MCA were small. We confirmed that, when analyzing images, it was difficult to distinguish the components of small-diameter vessels such as the BA and MCA other than the IPH, unless it was a severe stenosis condition, even if HR-MRI was used. In the future, a novel tool with high inplane spatial resolution and increased signal-to-noise ratio might allow for quantifying plaque features, including IPH. Lastly, the sample was small because not many patients had undergone HR-MRI as a follow-up procedure, which resulted in the wide ranges in the confidence intervals in the progression and regression of IPH from the multivariate analysis of progression and regression of stenosis (Table 4). The possibility of selective bias exists because it was a retrospective study. These limitations can be overcome by conducting large-scale, prospective, and long-term follow-up imaging analysis.

## 5. Conclusions

Despite these limitations, the results of this study are meaningful because they provided our consideration of the characteristics of atherosclerotic plaque components over time by using HR-MRI, and the differences between the progression and regression of plaques according to the progression and regression of IPH. The results of this study present an opportunity to discuss the risks related to atherosclerosis in patients, and the need for changing treatment strategies and lifestyle according to them by confirming the association between changes in plaque components and the risk factors for atherosclerosis that affect them.

## Figures and Tables

**Table 1:** Clinical characteristics of the subjects.

	Subjects (*n* = 42)
Age, years	63.10 ± 9.15
Male sex (%)	35 (83.3)
Interval of HR-MRI (months)	16.88 ± 12.53
Distribution of artery stenosis	
Basilar artery (%)	9 (21.4)
Proximal ICA (%)	26 (61.9)
Middle cerebral artery (%)	7 (16.7)
Intracranial artery stenosis (%)	16 (38.1)
Intraplaque hemorrhage (IPH) (%)	20 (47.6)
Stroke or TIA (%)	24 (57.1)
Symptomatic stenosis (%) *	16 (38.1)
Antiplatelet agent use	
No antiplatelet (%)	4 (9.5)
Mono antiplatelet (%)	17 (40.5)
Dual antiplatelet (%)	21 (50.0)
Statin intensity	
No statin (%)	2 (4.8)
Moderate intensity (%)	27 (64.3)
High intensity (%)	13 (30.9)
Hypertension (%)	32 (76.2)
Diabetes mellitus (DM) (%)	19 (45.2)
Uncontrolled DM (%) †	7 (36.8)
Dyslipidemia (%)	9 (21.4)
Coronary artery disease (%)	6 (14.3)
Atrial fibrillation (%)	2 (4.8)
Previous stroke (%)	3 (7.1)
Smoking (%)	14 (33.3)
Alcohol drinking (%)	19 (45.2)

Results are expressed as number (%) or mean ± standard deviation. HR-MRI, high-resolution magnetic resolution imaging; ICA, internal carotid artery; TIA, transient ischemic attack. * Stenosis in the cerebral artery, either intracranial or extracranial, leading to neurological symptoms of stroke or TIA. † Drug-resistant or in a patient with classic symptoms of hyperglycemia or hyperglycemic crisis, a random plasma glucose level ≥ 200 mg/dL.

**Table 2:** Comparison of the demographics of patients according to regression in cerebral artery stenosis.

	Stenosis without Regression (*n* = 27)	Stenosis with Regression (*n* = 15)	*p*-Value
Male	23 (85.2)	12 (80.0)	0.666
Age, years	65.70 ± 9.08	58.40 ± 7.43	0.011
Interval of HR-MRI (months)	16.26 ± 10.85	18.00 ± 15.45	0.671
Location (each artery, 100%)			
Basilar artery (*n* = 9)	6 (66.7)	3 (33.3)	
Proximal ICA (*n* = 26)	20 (76.9)	6 (23.1)	0.009
Middle cerebral artery (*n* = 7)	1 (14.3)	6 (85.7)	
Intracranial artery stenosis	7 (25.9)	9 (60.0)	0.029
Symptomatic stenosis *	11 (40.7)	5 (33.3)	0.636
Intraplaque hemorrhage (IPH) Regression of IPH	14 (51.9) 3 (11.1)	6 (40.0) 6 (40.0)	0.461 0.029
Stroke or TIA	13 (48.1)	11 (73.3)	0.114
Antiplatelet agent use			
No antiplatelet	4 (14.8)	0 (0)	
Mono antiplatelet	12 (44.4)	5 (33.3)	0.149
Dual antiplatelet	11 (40.7)	10 (66.7)	
Statin intensity			
No statin Moderate intensity High intensity	2 (7.4) 15 (55.6) 10 (37.0)	0 (0) 12 (80.0) 3 (20.0)	0.233
Hypertension Diabetes mellitus (DM)	21 (77.8) 12 (44.4)	11 (73.3) 7 (46.7)	0.746 0.89
Uncontrolled DM †	6 (50.0)	1 (14.3)	0.173
Dyslipidemia	7 (25.9)	2 (13.3)	0.341
Coronary artery disease	4 (15.4)	2 (13.3)	0.858
Atrial fibrillation	2 (7.7)	0 (0)	0.305
Smoking	12 (44.4)	2 (13.3)	0.04
Alcohol drinking	13 (50.0)	6 (40.0)	0.536
Previous stroke	2 (7.7)	1 (6.7)	0.903
Laboratory test			
Hb	11.34 ± 5.47	12.59 ± 4.59	0.459
WBC (×1000)	7.49 ± 2.37	8.26 ± 1.87	0.309
PLT (×1000)	216.96 ± 65.30	243.43 ± 62.69	0.233
HbA1c (initial) ‡	7.28 ± 1.24	8.30 ± 1.05	0.084
HbA1c (follow up) ‡	7.68 ± 1.72	6.70 ± 0.34	0.08
Change of HbA1c	0.4 ± 1.34	−1.60 ± 1.05	0.004
FDP	2.09 ± 1.28	1.58 ± 1.85	0.412
D-dimer	0.42 ± 0.31	0.31 ± 0.19	0.266
BUN	17.63 ± 6.44	16.36 ± 5.36	0.539
Creatinine	0.78 ± 0.45	0.76 ± 0.29	0.842
GFR	82.56 ± 18.30	90.16 ± 15.81	0.266
Uric acid	6.04 ± 1.58	4.52 ± 0.54	0.055
Total cholesterol	168.04 ± 33.14	172.79 ± 40.17	0.696
Triglyceride	178.43 ± 172.57	151.58 ± 77.93	0.614
High-density lipoprotein (HDL)	41.05 ± 9.39	41.00 ± 8.77	0.989
Low-density lipoprotein (LDL)	102.77 ± 28.19	111.85 ± 30.94	0.381

Results are expressed as number (%) or mean ± standard deviation. HR-MRI, high-resolution magnetic resolution imaging; ICA, internal carotid artery; TIA, transient ischemic attack. * Stenosis in the cerebral artery, either intracranial or extracranial, leading to neurological symptoms of stroke or TIA. † Drug-resistant or in a patient with classic symptoms of hyperglycemia or hyperglycemic crisis, random plasma glucose level ≥ 200 mg/dL. ‡ Stenosis without regression (*n* = 12) and stenosis with regression (*n* = 7).

**Table 3:** Comparison of the demographics of patients according to progression in cerebral artery stenosis.

	Stenosis without Progression (*n* = 35)	Stenosis with Progression (*n* = 7)	*p*-Value
Male	29 (82.9)	6 (85.7)	0.853
Age, years	61.86 ± 9.14	69.29 ± 6.65	0.048
Interval of HR-MRI (months)	16.83 ± 13.27	17.14 ± 8.59	0.953
Location (each artery, 100%)			
Basilar artery (*n* = 9)	8 (88.9)	1 (11.1)	
Proximal ICA (*n* = 26)	20 (76.9)	6 (23.1)	0.306
Middle cerebral artery (*n* = 7)	7 (100.0)	0 (0)	
Intracranial artery stenosis	15 (42.9)	1 (14.3)	0.222
Symptomatic stenosis *	12 (34.3)	4 (57.1)	0.397
Intraplaque hemorrhage (IPH) Progression of IPH	13 (37.1) 1 (2.9)	7 (100) 6 (85.7)	0.003 <0.001
Stroke or TIA	20 (57.1)	4 (57.1)	1
Antiplatelet agent use			
No antiplatelet	3 (8.6)	1 (14.3)	
Mono antiplatelet	14 (40.0)	3 (42.9)	0.862
Dual antiplatelet	18 (51.4)	3 (42.9)	
Statin intensity			
No statin Moderate intensity High intensity	1 (2.9) 25 (71.4) 9 (25.7)	1 (14.3) 2 (28.6) 4 (57.1)	0.077
Hypertension Diabetes mellitus (DM)	25 (71.4) 14 (40.0)	7 (100) 5 (71.4)	0.105 0.214
Uncontrolled DM †	4 (28.6)	3 (60.0)	0.211
Dyslipidemia	6 (17.1)	3 (42.9)	0.13
Coronary artery disease	4 (11.8)	2 (28.6)	0.268
Atrial fibrillation	2 (6.2)	0 (0)	1
Smoking	11 (31.4)	3 (42.9)	0.668
Alcohol drinking	18 (51.4)	1 (16.7)	0.191
Previous stroke	3 (8.8)	0 (0)	1
Laboratory test			
Hb	11.69 ± 5.35	12.32 ± 4.29	0.771
WBC (×1000)	7.91 ± 2.17	7.26 ± 2.39	0.491
PLT (×1000)	230.50 ± 62.85	211.86 ± 75.79	0.501
HbA1c (initial) ‡	7.90 ± 1.25	6.96 ± 1.06	0.154
HbA1c (follow up) ‡	6.97 ± 1.24	8.28 ± 1.67	0.08
Change of HbA1c	−0.93 ± 1.13	1.32 ± 1.49	0.003
FDP	1.76 ± 1.58	2.63 ± 0.81	0.299
D-dimer	0.32 ± 0.19	0.69 ± 0.46	0.206
BUN	16.58 ± 5.79	19.71 ± 6.82	0.218
Creatinine	0.77 ± 0.40	0.81 ± 0.38	0.816
GFR	86.68 ± 18.50	78.69 ± 13.67	0.298
Uric acid	5.19 ± 1.41	6.78 ± 1.34	0.053
Total cholesterol	165.03 ± 34.92	190.85 ± 31.75	0.081
Triglyceride	169.00 ± 153.03	190.86 ± 31.75	0.081
High-density lipoprotein (HDL)	40.82 ± 9.27	41.86 ± 8.69	0.791
Low-density lipoprotein (LDL)	101.96 ± 30.37	122.86 ± 15.88	0.09

Results are expressed as number (%) or mean ± standard deviation. HR-MRI, high-resolution magnetic resolution imaging; ICA, internal carotid artery; TIA, transient ischemic attack. * Stenosis in the cerebral artery, either intracranial or extracranial, leading to neurological symptoms of stroke or TIA. † Drug-resistant or in a patient with classic symptoms of hyperglycemia or hyperglycemic crisis, random plasma glucose level ≥ 200 mg/dL. ‡ Stenosis without progression (*n* = 14) and stenosis with progression (*n* = 5).

**Table 4:** Univariate and multivariate analyses of parameters associated with regression and progression of cerebral artery stenosis.

<Regression>				
	Crude OR (95% CI)	*p* Value	Adjusted OR (95% CI)	*p* Value
Age	0.90 (0.83–0.98)	0.019	0.87 (0.79–0.97)	0.014
Regression of IPH	5.33 (1.09–25.99)	0.038	10.13 (1.31–78.57)	0.027
Smoking	0.19 (0.04–1.02)	0.053	0.11 (0.01–0.83)	0.033
**<Progression>**				
	**Crude OR (95% CI)**	***p* Value**	**Adjusted OR (95% CI)**	***p* Value**
Age	1.10 (0.99–1.22)	0.061	1.01 (0.84–1.23)	0.897
Progression of IPH	204.00 (11.17–3724.26)	<0.001	115.80 (3.77–3554.18)	0.007
LDL	1.03 (0.99–1.06)	0.1	1.02 (0.96–1.09)	0.495

Results are expressed as odds ratios (ORs) and 95% confidence intervals (CIs). Variables with *p* < 0.1 by univariate analysis were entered into the multivariate analysis model. CI, confidence interval; IPH, intraplaque hemorrhage; OR, odds ratio. Results are expressed as odds ratios (ORs) and 95% confidence intervals (CIs). Variables with *p <* 0.1 by univariate analysis were entered into the multivariate analysis model. CI, confidence interval; IPH, intraplaque hemorrhage; LDL, low-density lipoprotein; OR, odds ratio.

## Data Availability

Clinical data may be provided to the editors upon appropriate request.
